# Discrete Neural Correlates for the Recognition of Negative Emotions: Insights from Frontotemporal Dementia

**DOI:** 10.1371/journal.pone.0067457

**Published:** 2013-06-21

**Authors:** Fiona Kumfor, Muireann Irish, John R. Hodges, Olivier Piguet

**Affiliations:** 1 Neuroscience Research Australia, Sydney, Australia; 2 School of Medical Sciences, the University of New South Wales, Sydney, Australia; 3 ARC Centre of Excellence in Cognition and its Disorders, the University of New South Wales, Sydney, Australia; 4 School of Psychology, the University of New South Wales, Sydney, Australia; University College London, United Kingdom

## Abstract

Patients with frontotemporal dementia have pervasive changes in emotion recognition and social cognition, yet the neural changes underlying these emotion processing deficits remain unclear. The multimodal system model of emotion proposes that basic emotions are dependent on distinct brain regions, which undergo significant pathological changes in frontotemporal dementia. As such, this syndrome may provide important insight into the impact of neural network degeneration upon the innate ability to recognise emotions. This study used voxel-based morphometry to identify discrete neural correlates involved in the recognition of basic emotions (anger, disgust, fear, sadness, surprise and happiness) in frontotemporal dementia. Forty frontotemporal dementia patients (18 behavioural-variant, 11 semantic dementia, 11 progressive nonfluent aphasia) and 27 healthy controls were tested on two facial emotion recognition tasks: The Ekman 60 and Ekman Caricatures. Although each frontotemporal dementia group showed impaired recognition of negative emotions, distinct associations between emotion-specific task performance and changes in grey matter intensity emerged. Fear recognition was associated with the right amygdala; disgust recognition with the left insula; anger recognition with the left middle and superior temporal gyrus; and sadness recognition with the left subcallosal cingulate, indicating that discrete neural substrates are necessary for emotion recognition in frontotemporal dementia. The erosion of emotion-specific neural networks in neurodegenerative disorders may produce distinct profiles of performance that are relevant to understanding the neurobiological basis of emotion processing.

## Introduction

Frontotemporal dementia (FTD) is a progressive neurodegenerative brain disorder associated with marked changes in social and emotion processing [1]–[9]. FTD encompasses three clinical subtypes that present with either changes in behaviour (behavioural-variant FTD (bvFTD)), or changes in language (semantic dementia (SD) and progressive nonfluent aphasia (PNFA)) [10], [11]. The clinical features observed in FTD subtypes reflect their underlying focal brain atrophy in the initial stages of the disease, namely the orbitomesial frontal regions in bvFTD; the anterior temporal lobes in SD (greater on one side, more commonly the left hemisphere); and the left insula, superior temporal gyrus and inferior frontal regions surrounding the Sylvian fissure in PNFA [12]–[16].

Despite their differing clinical presentations and associated brain atrophy, all three phenotypes experience a degree of emotion processing impairment, which is present on tasks using facial, auditory and film stimuli [2], [5]–[7], [9]. Typically, recognition of negative emotions is affected, whereas recognition of positive emotions remains relatively intact [1], [17]. General emotion recognition deficits in FTD have been associated with atrophy of the amygdala [5], [9], orbitofrontal and medial prefrontal cortex [5], [9], [18], [19], complementing previous reports in healthy adults [20]–[23]. Knowledge of neural correlates necessary for recognition of specific emotions in FTD subtypes remains, however, limited.

Some emotions, known as “basic emotions” (anger, disgust, fear, happiness, sadness, surprise), are proposed to be innate [24], [25]. They have evolved over time across species and are thought to rely on, at least partly, dissociable brain regions [22], [25]–[28]. Accordingly, a neurobiological model of emotion processing has been developed, referred to as the multimodal system model of emotion [2], [29]. Converging research from animal and human (lesion and imaging) studies indicates that processing of some basic emotions maps onto discrete brain regions, irrespective of the type of stimulus presented [28]–[30].

Evidence for a discrete multimodal system model of emotion is strongest for two basic emotions: fear and disgust. The amygdala is consistently implicated during fear conditioning in animals [31] and shows increased activation in humans when viewing fearful faces [32], [33]. Further, selective deficits in fear recognition have been identified in patients with bilateral amygdala damage, supporting the view that this structure is crucial for processing of fear [30], [34]–[36]. In contrast, disgust recognition is associated with insula and basal ganglia integrity in humans [29], [37]–[40]. The central role of these structures in processing signals of disgust is demonstrated by the disproportionate impairment of disgust recognition observed in patients with Huntington’s disease, or following focal damage to the insula and putamen [29], [41]. Evidence supporting the existence of specialised brain regions for processing the other basic emotions is less well established. Medial and orbitofrontal regions appear to be involved in processing of anger [42], [43], although selective impairment of anger recognition following lesions involving these brain regions has not been reported to date. The involvement of discrete neural substrates for processing of sadness, surprise and happiness also remain poorly understood [28]. Importantly, these emotion-specific regions, namely the amygdala, insula and frontal cortex, are brain regions showing significant neuronal loss and abnormal protein deposition in FTD [15]. As such, in addition to providing insight into how specific lesions can cause impairment, studies of patients with neurodegenerative conditions can inform how specific brain regions form networks that are central to supporting different cognitive functions [44].

This study aimed to test the multimodal system model of emotion and investigate the contribution of dissociable neural substrates to the recognition of basic emotions (anger, disgust, fear, sadness, happiness and surprise) in FTD, using voxel-based morphometry; an unbiased, automated, whole-brain structural analysis method. In order to identify emotion-specific neural correlates in FTD, this study used both a traditional facial emotion recognition task (Ekman 60) and a modified facial emotion recognition task (Ekman Caricatures). On the Ekman Caricatures task the intensity of the emotion expressed is enhanced, thereby reducing the attentional and perceptual demands of the task [45], [46]. Using the Ekman Caricatures task, a recent study showed that emotion recognition deficits observed in bvFTD and PNFA could be alleviated to some extent by increasing the intensity of the emotional expression [7]. In contrast, increasing the emotional intensity of facial expressions did not improve performance in SD, suggesting that, in these patients, reduced emotion recognition is due to a primary emotion processing impairment. In bvFTD and PNFA, however, poor emotion recognition performance may be in part mediated by attentional or perceptual demands of the task [7].

By using two tasks of facial emotion recognition, which vary in attentional and perceptual task demands, we aimed to identify common neural correlates associated with basic emotion identification, irrespective of task demands. Based on the multimodal system model we hypothesised that fear recognition would be dependent on amygdala integrity, and disgust recognition would be dependent on insula integrity, on both emotion recognition tasks. No specific hypotheses for neural substrates associated with processing the basic emotions anger, sadness, surprise or happiness were made, as evidence for specific neural substrates for processing of these emotions is mixed. In doing so, we aimed to determine the relative importance of specific neural structures for the recognition of basic human emotions in FTD, and establish the crucial role specific neural structures play in the recognition of basic emotions.

## Materials and Methods

### Participants

Forty FTD patients (18 bvFTD, 11 SD and 11 PNFA) were compared with 27 healthy controls. Patients were recruited from FRONTIER, the frontotemporal dementia clinical research group based at Neuroscience Research Australia, Sydney. All patients underwent clinical assessment, cognitive neuropsychological assessment and structural magnetic resonance imaging (MRI). An experienced behavioural neurologist assessed all patients and diagnosis was established by consensus between the neurologist, neuropsychologist and occupational therapist, based on extensive clinical investigations, cognitive assessment, and evidence of atrophy on structural MRI brain scans. All patients met current clinical diagnostic criteria [10], [11]. In brief, the bvFTD group presented with changes in behaviour and personality, displaying emotional blunting, loss of insight, and reduced motivation. The SD group presented with reduced semantic knowledge, demonstrated by impaired naming, and comprehension, in the context of relatively preserved phonology and syntax. The PNFA group presented with impaired expressive language characterised by effortful speech, phonetic distortions and impaired grammatical structure, in the context of relatively intact comprehension.

Healthy control participants were recruited from the local area and included family members of the patients and individuals recruited from local community clubs. All control participants underwent neuropsychological assessment, and structural MRI. For all participants, exclusion criteria included: concurrent psychiatric disturbance, other types of dementia or other neurological disease, including cerebrovascular disease, history of substance abuse and/or use of medications with central nervous system side effects and for controls, an Addenbrooke’s Cognitive Examination – Revised (ACE-R) score below 88/100 [47].

The behavioural performance for 29 of the 40 FTD patients (10/18 bvFTD, 10/11 SD, 9/11 PNFA) has been previously reported in a study investigating the effects of increasing emotional intensity on emotion recognition in FTD [7]. The current study, however, sought to establish the neural correlates of recognition of specific basic emotions in FTD.

### Ethics Statement

This research was conducted in accordance with the Declaration of Helsinki. Ethics approval was obtained from the Human Research Ethics Committee of South Eastern Sydney/Illawarra Area Health Service (HREC 10/126) and the University of New South Wales Human Research Ethics Advisory panel D (Biomedical, ref. #10035). Participant or family written consent was obtained from each participant. Participants volunteered their time and were reimbursed for travel costs.

### Behavioural testing

#### General Cognitive Tests

Participants were assessed on the ACE-R [47] as a measure of general cognitive ability. In addition, all participants completed tests of attention (Digit Span Forwards subtest of the Wechsler Adult Intelligence Scale [48] and Trail Making Test A [49]), visuospatial ability (Rey Complex Figure [50]), confrontation naming (Sydney Language Battery, naming subscale [51]) and face perception (Face Matching Task [3]). These tests were administered as part of a larger cognitive assessment battery.

#### Facial Emotion Recognition Tests

Integrity of facial emotion recognition was measured using the Ekman 60 [52], [53] and the Ekman Caricatures [52] tasks. On both tasks, participants viewed faces expressing one of six basic emotions (anger, disgust, fear, sadness, surprise, happiness), on a computer screen, one at a time, in a pseudorandom order. Images were presented for 5 sec and participants were instructed to determine the emotional label (*anger, disgust, fear, sadness, surprise* or *happiness*) that best matched the facial emotion being displayed. Understanding of the emotional labels was confirmed prior to the task and participants unable to understand the labels were excluded from the study. Participants had unlimited time to respond. For the Ekman 60, the computer automatically recorded the participant’s response, whereas for the Caricatures task, the researcher recorded the response. For both tasks, participants responded by using the mouse, pointing or saying their response. The emotional labels remained on the screen until the participant responded. No feedback was provided.

Stimuli included in the Ekman 60 were photographs of natural facial expressions (100% emotional intensity), 10 exemplars per emotion. On the Caricatures task, the stimuli were expressions of two models (MO and JJ) from the Ekman 60 task that have been digitally manipulated to increase the emotional intensity of the natural expression by +15%, +30%, +50% or +75%, resulting in 8 exemplars per emotion [52]. For the purposes of this study, scores for the Caricatures task were derived by averaging across the four levels of intensity, for each basic emotion.

### Image acquisition

All participants underwent whole brain magnetic resonance imaging (MRI) with a 3-Tesla (3-T) Phillips MRI scanner with standard quadrature head coil (8 channels). High resolution T1-images were obtained in the coronal plane using the following protocol: 256×256 matrix, 200 slices, 1 mm^3^ isotropic voxels, echo time/repetition time  =  2.6/5.8 ms, flip angle α  = 19°.

### Data preprocessing

MRI data were analysed using FSL-voxel-based morphometry [54]–[56], part of the FMRIB software library package (http://www.fmrib.ox.ac.uk/fsl/fslvbm/index.html; [57].) Structural images were brain-extracted using BET, following which, tissue segmentation was conducted using FMRIB’s automatic segmentation tool (FAST) [58]. Grey matter partial volume maps were aligned to Montreal Neurological Institute standard space (MNI152) using non-linear registration (FNIRT) [59], [60], which uses a *b-*spline representation of the registration warp field [61]. A study-specific template was created and the native grey matter images were then non-linearly re-registered. Modulation of the registered partial volume maps was carried out (to correct for local expansion or contraction), by dividing them by the Jacobian of the warp field. Note that the modulation did not include the affine part of the registration so that participants were matched for brain size. The modulated, segmented images were smoothed with an isotropic Gaussian kernel with a sigma of 3mm (FWHM: 8 mm).

### Behavioural analyses

Data were analyzed using SPSS version 20.0 (IBM, Inc., Chicago, IL, USA). Between-group comparisons for each of the relevant demographic and neuropsychological variables were performed using univariate analysis of variance (ANOVA) or, where appropriate, analyses of covariance for continuous measures, and chi-square tests for categorical measures. Recognition of each basic emotion was investigated for the two facial emotion recognition tasks. For each task, a 6×4 repeated measures ANOVA was conducted with Emotion (Anger, Disgust, Fear, Sadness, Surprise, Happiness) as the repeated, within subjects variable and Diagnosis as the between subjects variable (Control, bvFTD, SD, PNFA). Effect sizes using partial eta squared (η_p_^2^) were calculated for main and interaction effects. Follow-up *post hoc* analyses (Bonferroni correction) were conducted to investigate differences across groups for each individual emotion.

### Voxel-based morphometry analyses

A voxel-wise general linear model was applied to investigate grey matter intensity differences, using permutation-based, non-parametric statistics, with 5000 permutations per contrast [62]. Differences in cortical grey matter intensities between each patient group (bvFTD, SD, PNFA) and controls were assessed using t-tests. Age was included as a nuisance variable for all contrasts. For the atrophy analyses, the statistical threshold was set at *p*<.05, fully corrected for multiple comparisons [Family Wise Error (FWE)].

Next, correlations between behavioural performances, specific to each emotion type, on the two emotion recognition tasks were conducted. Two separate sets of identical contrasts were employed across both versions of the emotion recognition tasks (Ekman 60, Ekman Caricatures) for each emotion type (Anger, Disgust, Fear, Sadness, Surprise, Happiness). Firstly, all 6 emotions were entered simultaneously into the design matrix. Then, specific contrasts to investigate the neural correlates of each basic emotion independent of the others were run concurrently (e.g., for six emotions, neglecting nuisance variables the contrasts were: anger [1,0,0,0,0,0], disgust [0,1,0,0,0,0,], fear [0,0,1,0,0,0], sadness [0,0,0,1,0,0], surprise [0,0,0,0,1,0], happiness [0,0,0,0,0,1]). This approach allowed us to investigate the unique associations between grey-matter intensity and performance for each emotion of interest while covarying for performance on each of the other emotions (see [18]). Age was included as a nuisance variable, for all contrasts. Correlations between emotion recognition performance in each emotion subtype and regions of grey matter intensity were investigated in bvFTD, SD and PNFA subtypes and healthy controls combined. This method has been adopted in previous studies including FTD subtypes [63], [64] and serves to achieve greater variance in behavioural scores, thus increasing the statistical power to detect brain-behaviour relationships. For the behavioural analyses, no significant associations were present following correction for multiple comparisons using FWE. Therefore, for all behavioural analyses, the significance threshold was set at *p*<.001, uncorrected for multiple comparisons. An additional conservative cluster extent threshold of 100 contiguous voxels was used to reduce the likelihood of false positive voxels [65], [66]. This approach reduces the level of Type I errors while mitigating the risks of Type II errors [67].

Anatomical locations of significant results were overlaid on the MNI standard brain, with maximum coordinates provided in MNI stereotaxic space. Anatomical labels were determined with reference to the Harvard-Oxford probabilistic cortical and subcortical atlases.

## Results

### Demographic and Background Neuropsychological Data

Groups were matched for sex, age and education (all *p* values >.05) (Table 1). Disease duration differed across patient groups, with SD having longer disease duration than PNFA. This difference reflects the generally longer time to diagnosis in SD compared to other FTD subtypes. On the general cognitive screening measure ACE-R, an overall effect of diagnosis was present, with all patient groups performing worse than controls. The SD group scored lower than the bvFTD and PNFA groups, reflecting the semantic language demands on this task. Cognitive testing revealed neuropsychological profiles that were generally characteristic of each patient group. BvFTD showed poorer attention compared to controls on the Trail Making Test A and poor visuoconstruction on the Rey Complex Figure copy, indicating reduced planning and organisation on this task. BvFTD also showed some reductions in confrontation naming, although to a lesser extent than the SD group, and poorer simple face processing, compared to controls. The SD group was significantly impaired on the naming task, but performed within normal limits on tasks of attention and visuospatial functioning, consistent with their clinical presentation. The PNFA group performed poorly on tasks of auditory attention and naming, which reflects their reduced expressive language output. Processing speed, based on their performance on the Trail Making Test A, was also reduced compared to controls (Table 1).

**Table 1:** Demographics and neuropsychological data for healthy controls and frontotemporal dementia subtypes.

	Controls	bvFTD	SD	PNFA	F	*p*	*Post hoc*
Sex (M/F)	16/11	13/5	7/4	6/5	1.2†	ns	
Age (years)	64.3 (3.7)	63.8 (8.2)	62.4 (9.3)	64.8 (10.0)	0.2	ns	
Education (years)	13.6 (2.1)	11.9 (3.3)	13.3 (3.4)	11.9 (3.2)	1.8	ns	
Disease Duration (months)	n/a	46.8 (26.6)	65.2 (28.2)	27.3 (8.4)	7.0	*	PNFA < SD
ACE-R	95.7 (3.3)	75.4 (12.4)	53.9 (31.9)	72.0 (18.5)	36.7	**	Patients < Controls; SD < bvFTD, PNFA
Digits forwards (max span)^#^	7.0 (1.3)	6.1 (1.1)	6.0 (1.3)	4.4 (1.2)	11.5	**	PNFA < Controls
Trails A	31.0 (11.1)	54.4 (18.1)	37.9 (19.0)	48.5 (16.8)	9.2	**	bvFTD, PNFA < Controls
RCF Copy	33.2 (3.0)	28.5 (5.6)	32.7 (4.3)	29.7 (5.7)	4.4	**	bvFTD < Controls
Naming^#^	27.0 (2.3)	21.2 (5.2)	5.0 (2.7)	19.7 (6.8)	72.1	**	Patients < Controls; SD < bvFTD, PNFA
Face Matching^§^	36.1 (4.0)	28.5 (7.9)	33.6 (7.0)	32.4 (5.5)	5.486	*	bvFTD < Controls

*Note.* ** *p*<.01; * *p*<.05; ns *p*>.05. Values are: Mean (standard deviation). n/a  =  not applicable. Abbreviations: bvFTD  =  behavioural-variant frontotemporal dementia, SD  =  semantic dementia, PNFA  =  progressive nonfluent aphasia, ACE-R  =  Addenbrooke’s Cognitive Examination-Revised, RCF  =  Rey Complex Figure. †  =  χ^2^. ^#^ Score missing for one PNFA participant.^ §^ Scores missing for two bvFTD and two PNFA participants.

### Emotion Recognition: Behavioural data

On the Ekman 60, a significant effect of diagnosis was present (*F*(3,63) = 17.6, *p*<.001, η_p_^2^ = .456), and a significant main effect of emotion was also observed (*F*(1,63) = 50.0, *p*<.001, η_p_^2^ = .443). Importantly, an interaction between emotion and diagnosis was present (*F*(13,263) = 3.0, *p*<.001, η_p_^2^ = .126). *Post hoc* analyses revealed a significant effect of diagnosis for all negative emotions. A significant effect of diagnosis was also seen for happiness, although performance across all groups approached ceiling for this emotion (i.e., above 90% for patients and controls) (Table 2). *Post hoc* analyses investigating the performance of each diagnostic group for each basic emotion revealed that bvFTD and SD performed poorer than controls for all negative emotions, while PNFA showed significant deficits with respect to controls for the recognition of anger, fear and sadness. BvFTD, SD and PNFA did not perform significantly differently from each other for any of the specific emotions (Table 2).

**Table 2:** Performance on the Ekman 60 and Caricatures tasks, across the six basic emotions for healthy controls and frontotemporal dementia subtypes.

	Controls	bvFTD	SD	PNFA	F	p	Post hoc
**Ekman 60**							
Anger	82.6 (15.6)	57.8 (20.2)	60.0 (26.1)	56.4 (20.1)	8.6	**	Patients < Controls
Disgust	85.6 (14.8)	56.7 (28.3)	51.8 (19.9)	72.7 (19.5)	10.4	**	bvFTD, SD < Controls
Fear	71.1 (20.4)	28.3 (12.9)	43.6 (31.7)	49.1 (28.8)	13.6	**	Patients < Controls
Sadness	83.3 (10.0)	59.4 (18.6)	59.1 (34.5)	61.8 (28.6)	6.5	**	Patients < Controls
Surprise	85.2 (14.0)	74.4 (26.0)	69.1 (25.9)	71.8 (27.1)	2.0	ns	
Happiness	99.6 (1.9)	95.6 (6.2)	94.5 (5.2)	96.4 (9.2)	3.3	*	ns
**Caricatures**							
Anger	94.9 (10.0)	67.4 (27.5)	59.1 (30.2)	81.8 (24.0)	9.5	**	bvFTD, SD < Controls
Disgust	96.8 (8.9)	68.8 (29.2)	46.6 (35.4)	88.6 (18.1)	14.9	**	bvFTD, SD < Controls; SD < PNFA
Fear	88.0 (15.3)	33.3 (23.9)	43.2 (32.8)	72.7 (34.4)	20.8	**	bvFTD, SD < Controls; bvFTD, SD < PNFA
Sadness	93.1 (14.4)	58.3 (36.1)	58.0 (34.1)	83.0 (29.7)	7.7	**	bvFTD, SD < Controls
Surprise	88.9 (17.1)	75.0 (33.5)	71.6 (28.0)	83.0 (25.2)	1.7	ns	
Happiness	99.5 (2.4)	95.1 (15.5)	100.0 (0.0)	94.3 (15.2)	1.3	ns	

*Note.* ** *p*<.01; * *p*<.05; ns *p*>.05. Scores are percentage correct: Mean (standard deviation). bvFTD  =  behavioural-variant frontotemporal dementia, SD  =  semantic dementia, PNFA  =  progressive nonfluent aphasia.

On the Caricatures task, a significant effect of diagnosis was present (*F*(3,63) = 15.0, *p*<.001, η_p_^2^ = .416) and a main effect of emotion was also observed (*F*(4,266) = 27.9, *p*<.001, η_p_^2^ = .307). Again, an interaction between diagnosis and emotion was evident (*F*(13, 266) = 5.593, *p*<.001, η_p_^2^ = .210). *Post hoc* analyses investigating the effect of diagnosis for each emotion, revealed a significant effect of diagnosis for each negative emotion only (Table 2). The bvFTD and SD group performed poorer than controls for all negative emotions, and worse than the PNFA group for recognition of fear. Recognition of disgust in SD was significantly worse than the PNFA group, and both bvFTD and SD performed poorer than PNFA for recognition of fear. No significant differences between PNFA and controls, or between bvFTD and SD were present for any of the specific emotions on this task (Table 2).

We also investigated the possible role of face perception and attention on emotion recognition performance, by repeating the same analyses for the Ekman 60 and Caricatures, including Face Matching and Digit Span Forwards (maximum span), as covariates. The interaction between Emotion and Diagnosis remained significant for both the Ekman 60 (*F*(15, 240) = 2.382, *p* = .005, η_p_^2^ = .111) and the Caricatures (*F*(15, 233) = 4.389, *p*<.001, η_p_^2^ = .188) tasks, although the size of the effect was smaller. Together, these results indicate that while impairments in attention and face perception ability contribute to poor emotion recognition performance in FTD, they do not account entirely for the emotion recognition deficits seen in these patients.

In summary, significant emotion recognition impairments were found for all negative emotions, across both emotion recognition tasks. Patients with bvFTD and SD displayed pervasive deficits across all negative emotions, whereas disgust was preserved in PNFA patients. In the bvFTD and SD groups, these deficits persisted even after increasing the emotional intensity of the stimuli, with both groups continuing to be significantly impaired across all negative emotions. In contrast, increasing the emotional intensity reduced the emotion processing difficulties in the PNFA group.

### Voxel-based morphometry - Group analysis

#### Patterns of atrophy

**Figure 1:**
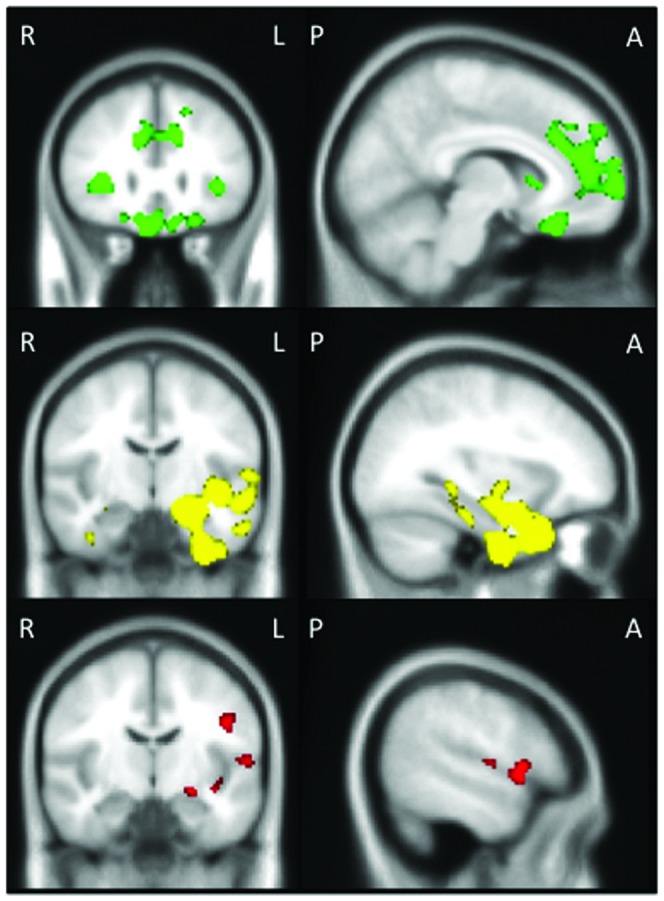
Regions of atrophy in behavioural-variant frontotemporal dementia, semantic dementia and progressive nonfluent aphasia. Voxel-based morphometry analyses showing brain regions of decreased grey matter intensity in behavioural-variant frontotemporal dementia (bvFTD): x = 8, y = 28, z = –28, *t*>3.73 (top); semantic dementia (SD): x = –32, y = –10, z = –50, *t*>3.73 (middle); and progressive nonfluent aphasia (PNFA): x = – 54, y = –10, z = 8, *t*>3.21 (bottom) relative to controls. Coloured voxels show regions that were significant in the analyses with *p*<.05 corrected for multiple comparisons (FWE). Age included as a nuisance variable for all contrasts. Clusters are overlaid on the standard Montreal Neurological Institute brain. R  =  right, L  =  left, P  =  posterior, A  =  anterior.

Patients with bvFTD showed grey matter intensity decrease in the frontal pole and orbitofrontal regions bilaterally, extending into the right parahippocampal and hippocampal regions, amygdala and thalamus, and the left medial prefrontal cortex, anterior cingulate and paracingulate regions, compared to controls (Figure 1). Patients with SD showed grey matter intensity decrease, primarily in the left temporal pole region, extending to the left orbitofrontal cortex, parahippocampal gyrus, amygdala, hippocampus, putamen, insula, middle and superior temporal gyrus, compared to controls. PNFA showed reduced grey matter intensity in the left inferior frontal gyrus, precentral gyrus and insula, compared to controls.

#### Neural Correlates of Emotion Recognition

**Figure 2:**
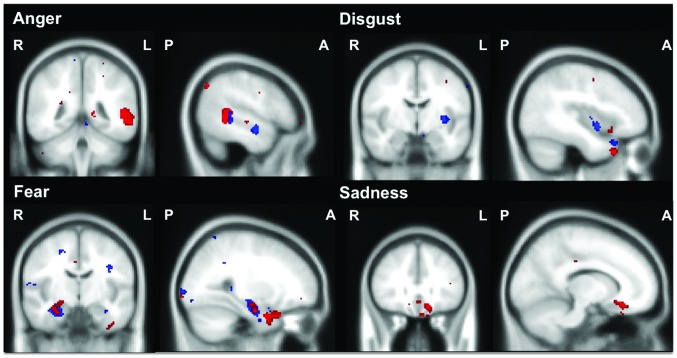
Brain regions correlated with negative emotion recognition on the Ekman 60 (Red) and Caricatures (Blue). Voxel-based morphometry analyses showing common brain regions in which grey matter intensity in all participants combined correlated significantly with emotion recognition for each basic emotion on the Ekman 60 (Red) and Caricatures (Blue) tasks. MNI coordinates: Anger: x = –52, y = –44, z = –6; Disgust: x = –40, y = –6, z = –32; Fear: x = 28, y = –10, z = –10; and Sadness: x = –12, y = 30, z = –16. Coloured voxels show regions that were significant in the analyses with *p*<.001 uncorrected for all contrasts. Age included as a nuisance variable for all contrasts. All clusters reported *t*>3.95. Clusters are overlaid on the Montreal Neurological Institute standard brain. R  =  right, L  =  left, P  =  posterior, A  =  anterior.

Anger: Ekman 60 performance was associated with grey matter intensity of the left superior and middle temporal gyrus, right precuneus, and left posterior parahippocampal gyrus and lateral occipital cortex. On the Caricatures task, a significant cluster in the left middle temporal gyrus, extending into the superior temporal gyrus and insula was also identified, along with a more extensive network of regions that included the left temporal pole, fusiform cortex, orbitofrontal cortex, parahippocampal gyrus, hippocampus and occipital pole (Table 3, Figure 2).

**Table 3:** Voxel-based morphometry analyses showing significant correlations between grey matter intensity and recognition of negative emotions on the Ekman 60 and Ekman Caricatures for all participant groups combined.

Regions		Hemisphere	MNI Coordinates			Number of Voxels
			X	Y	Z	
**ANGER**						
	**Ekman 60**					
Left middle temporal gyrus, extending into superior temporal gyrus		Left	–58	–48	–6	377
Lateral occipital cortex		Left	–50	–68	40	263
Cuneal cortex, precuneus		Right	20	–74	18	195
Parahippocampal gyrus		Left	–10	–36	–4	133
Postcentral gyrus, bordering precentral gyrus		Left	–44	–14	30	111
**Frontal pole**		Left	–36	38	2	102
	**Caricatures**					
Temporal pole, extending into left fusiform cortex, parahippocampal gyrus and hippocampus		Left	–36	6	–44	849
Orbitofrontal cortex		Left	–22	16	–26	505
Middle temporal gyrus,extending into superior temporal gyrus and insula		Left	–60	–6	–24	358
Occipital pole		Left	–18	–94	–12	141
Middle temporal gyrus, extending into superior temporal gyrus		Left	–50	–42	–4	128
Temporal pole		Right	40	24	–38	104
**DISGUST**						
	**Ekman 60**					
Insula, extending into orbitofrontal cortex		Left	–36	12	–18	149
Thalamus		Right	2	–2	–2	130
Temporal pole, orbitofrontal cortex, insula		Right	32	6	–20	119
Temporal pole		Left	–38	22	–42	118
	**Caricatures**					
Insula		Left	–42	0	–12	157
Temporal pole, orbitofrontal cortex		Left	–42	20	–28	122
Lateral occipital cortex		Left	–54	–64	8	104
**FEAR**						
	**Ekman 60**					
Inferior temporal gyrus extending into right temporal pole, orbitofrontal gyrus and parahippocampal gyrus		Right	40	2	–48	697
Paracingulate gyrus, extending into anterior cingulate		Left	–4	48	6	185
Hippocampus, extending into amygdala		Right	26	–12	–24	148
Temporal fusiform cortex		Left	–38	–16	–42	112
	**Caricatures**					
Subcallosal cortex, extending into medial frontal cortex and anterior cingulate		Right	4	26	–28	1068
Planum temporale		Right	40	–32	8	386
Putamen		Right	22	16	–6	332
Frontal pole		Right	10	68	–6	322
Parahippocampal gyrus, hippocampus, amygdala		Right	28	–10	–28	315
Precuneus		Right	6	–68	60	138
Occipital pole		Right	22	–102	–6	136
Lingual gyrus, occipital pole		Left	–12	–88	–6	118
**SADNESS**						
	**Ekman 60**					
Subcallosal cortex		Left	–2	26	–28	207
	**Caricatures**					
No significant clusters						

*Note.* All results uncorrected at *p*<.001; only clusters with at least 100 contiguous voxels are reported. All clusters reported *t*>3.95. MNI  =  Montreal Neurological Institute. Age included as a nuisance variable for all contrasts.

Disgust: On the Ekman 60 task, a region in the left insula, extending into the orbitofrontal cortex was significantly associated with disgust recognition, along with regions in the right thalamus and the temporal poles bilaterally. On the Caricatures task, a region of grey matter intensity in the left insula was again associated with disgust recognition, as well as regions in the left temporal pole and orbitofrontal cortex. In addition, the left occipital cortex was also associated with disgust recognition on this task (Table 3, Figure 2).

Fear: Ekman 60 performance was associated with grey matter intensity in the right amygdala, hippocampus, and the left anterior cingulate, extending into the paracingulate gyrus. Regions in the right inferior temporal gyrus, right temporal pole, orbitofrontal cortex and parahippocampal gyrus, together with a region in the left temporal fusiform cortex was also associated with fear recognition on this task. Similarly on the Caricatures task, the right amygdala, hippocampus and anterior cingulate cortex were associated with fear recognition, along with a more extensive network of regions that included the planum temporale, Heschl’s gyrus, frontal cortex, including the subcallosal cortex, medial and superior frontal cortex and occipital regions including the occipital pole and the left lingual gyrus (Table 3, Figure 2).

**Figure 3:**
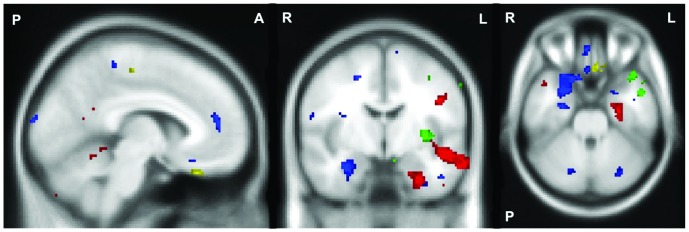
Dissociable brain regions associated with recognition of anger, disgust, fear and sadness. Voxel-based morphometry analyses showing dissociable brain areas in which grey matter intensity in all participants combined, correlated significantly with emotion recognition irrespective of task for the emotions: Anger (Red), Disgust (Green), Fear (Blue) and Sadness (Yellow). Montreal Neurological Institute coordinates: x = –6, y = –6, z = –28; Coloured voxels show regions that were significant in the analyses with *p*<.001 uncorrected for all contrasts. Age included as a nuisance variable for all contrasts. All clusters reported *t*>3.95. Clusters are overlaid on the Montreal Neurological Institute standard brain. P  =  posterior, A  =  anterior, R  =  right, L  =  left.

Sadness: Ekman 60 performance was associated with the left subcallosal cingulate cortex. No clusters larger than 100 contiguous voxels were associated with sadness recognition on the Caricatures task (Table 3, Figure 2). A summary of the distinct brain regions correlating with recognition of anger, disgust, fear and sadness is presented in Figure 3.

Recognition of positive emotions, happiness and surprise, were included in the design matrix, in order to take into account task performance across all basic emotions. No significant clusters were identified for recognition of surprise, which is consistent with the lack of group differences on performance for this emotion across both tasks. Performance for recognition of happiness was at ceiling in almost all participants, regardless of group membership. While small significant clusters were identified in the VBM analyses associated with happiness (Table S1), these findings are likely to have limited clinical meaning, given the small number of participants who committed any errors on this task.

## Discussion

This study has successfully established the existence of dissociable neural regions supporting recognition of the four basic negative emotions: fear, disgust, anger and sadness, emotions for which neural correlates have not been clearly identified in FTD. In keeping with our hypotheses, fear recognition was associated with the right amygdala, hippocampus, and anterior cingulate, whereas, disgust recognition was associated with the left insula and left temporal pole. In addition, this study also uncovered associations between anger recognition and the region surrounding the left superior temporal sulcus, and between sadness recognition and the subcallosal cingulate. Together these results provide strong support for the applicability of the multimodal system model of emotion for understanding the emotion processing deficits seen in FTD.

The right amygdala and anterior cingulate were the only regions involved in fear recognition, irrespective of task. The association between the right amygdala and fear recognition in FTD converges strongly with previous findings and demonstrates the integral nature of this structure for processing fear [31], [34], [35], [68]–[71]. The anterior cingulate cortex has previously been shown to activate during recognition of fearful expressions in healthy adults [70]. Our results provide additional support for the critical role of the anterior cingulate for processing fear signals. The concurrent involvement of these structures for processing fear-related stimuli likely reflects their strong bidirectional connectivity [72]–[74].

The ability to correctly identify facial stimuli conveying the emotion disgust was associated with the integrity of the insula, resonating with previous reports in the literature [37], [38], [40], [75]. Specialisation within the insula likely explains the relatively intact disgust recognition in PNFA despite the marked insula atrophy observed in this group. The anteroventral portion of the insula is specialised for emotion processing, whereas dorsal regions are predominantly associated with language expression [76], reflecting the language deficits associated with the burden of atrophy observed in PNFA. In contrast, the well-documented deficits in disgust recognition in Huntington’s disease indicate an involvement of ventral, emotion-processing, insular regions. Detailed investigations will be required to clarify the nature and functions of the insula subdivisions in cognitive and emotion processing [76], [77].

The left superior temporal sulcus was the single brain region identified by both tasks during processing of angry facial stimuli. In healthy individuals, regions surrounding the superior temporal sulcus activate when viewing angry faces, and when detecting anger from meaningless speech, providing converging support for the specialisation of this region in anger recognition [75], [78]. In contrast, sadness recognition was associated with subcallosal cingulate cortex integrity. This association mirrors functional imaging findings in healthy adults [70], [79], [80] and in depressed individuals [81], suggesting that abnormal changes in this region may not only affect facial emotion recognition, but may also contribute to depressed mood in some FTD patients. Evidence supporting the existence of specialised regions for recognition of sadness and anger is less well established than for fear and disgust. Our results provide important evidence for the role of the superior temporal sulcus and the subcallosal cingulate for the recognition of anger and sadness respectively.

**Figure 4:**
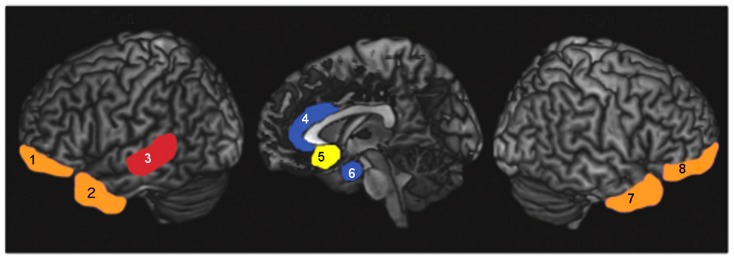
The multimodal system model of emotion. Proposed neural correlates associated with processing of basic emotions based on current and prior results from studies including frontotemporal dementia (FTD) patients. Anger (Red), Fear (Blue), Sadness (Yellow). Structures implicated in general emotion processing are shown in orange. 1: Left orbitofrontal cortex; 2: Left temporal pole; 3: Left superior temporal sulcus; 4: Anterior cingulate; 5: Subcallosal cingulate; 6: Right amygdala; 7: Right temporal pole, 8: Right orbitofrontal cortex. *Note.* Insula, which is implicated in the processing of disgust stimuli, is not visible on lateral or sagittal views. Neuroanatomical regions are approximate.

The novel findings of discrete neural correlates for the emotions fear, disgust, anger and sadness, in FTD, are consistent with the multimodal system model of emotion [2] (Figure 4). Consistent with previous research in FTD, our study also identified structures generally implicated in emotion recognition, including the orbitofrontal cortex and temporal regions [5], [9], [18], [19]. The role of the orbitofrontal cortex for recognition of multiple basic emotions is in keeping with its central role in emotion processing [82]–[86], while the temporal pole and the fusiform gyrus have been shown to play an important role in processing of social and emotional stimuli, and face perception [87], [88]. Together, our results demonstrate the presence of dissociable neural substrates specialised for the recognition of specific negative emotions, as well as the existence of structures important for general aspects of emotion processing. These findings demonstrate the utility of neurodegenerative disorders in testing models of complex behaviour, such as emotion recognition.

By combining two facial emotion recognition tasks that varied in the emotional intensity of the facial expression, we were able to determine the contribution of attentional and perceptual deficits to emotion recognition performance in FTD. Consistent with previous results, the behavioural analyses indicated that impairments in early cognitive processes (attention, visuospatial) do not explain fully, the profound emotion processing deficits observed in some FTD patients [7], [89]. Importantly, while this study successfully linked the recognition of basic negative emotions to specific brain structures, neural correlates underlying recognition of positive emotions remain elusive. The facts that severe deficits for the recognition of positive emotions are rare and that relatively few positive emotions can be portrayed via different facial expressions compared to negative emotions, probably account for this lack of evidence. Stimuli that can measure various facets of positive emotions and emotion recognition tasks of greater difficulty that will avoid ceiling effects are needed in order to detect high-level deficits in recognition of positive emotions [90], [91].

This study used FTD as a model to examine brain-behaviour relationships of emotion processing. While the number of patients may appear small, the sample size compares favourably with other studies investigating clinical features of neurodegenerative disorders. Results, however, need to be interpreted with this caveat in mind; for example, VBM analyses were not corrected for multiple comparisons (although a minimum cluster size of at least 100 voxels was applied to minimise spurious findings). This caution notwithstanding, the brain regions we identified are remarkably consistent with previous studies using fMRI, patient lesion and animal models, providing additional support for our results.

In summary, we have demonstrated the existence of emotion-specific brain regions necessary for the recognition of negative basic emotions. Damage to these structures produces marked impairments in primary emotion processing in FTD patients. Although previous studies in FTD have suggested that frontal and temporal lobe structures are important for emotion processing, this study is the first to confirm that dissociable brain regions are necessary for recognition of different basic emotions in FTD patients. In particular, associations between the amygdala and fear recognition and the insula and disgust recognition confirm that these brain regions are specialised for processing these emotion signal types. Importantly, by using two tasks of facial emotion recognition that varied in perceptual and attentional demands, we also successfully identified discrete neural regions associated with anger and sadness recognition, supporting the hypothesis that specialised brain regions also exist for processing these negative emotions. The ability to recognise emotions in others is a complex process. Here, we have shown that specialised neural regions across the frontal and temporal lobes are necessary for recognising different emotions, providing further support for the existence of “basic emotions” and improving our understanding of the neurobiological basis of emotion processing.

## Supporting Information

Table S1 **Voxel-based morphometry analyses showing significant correlations between grey matter intensity and recognition of happiness on the Ekman 60 and Ekman Caricatures tasks.** *Note.* All results uncorrected at *p*<.001; only clusters with at least 100 contiguous voxels are reported. All clusters reported *t*>3.95. MNI  =  Montreal Neurological Institute. No clusters greater than 100 voxels were significantly associated with surprise recognition on the Ekman 60 or Ekman Caricatures task. Age included as a nuisance variable for all contrasts.
(DOCX)
