# Supplementary material for: Discrete Neural Correlates for the Recognition of Negative Emotions: Insights from Frontotemporal Dementia
Source: PLoS One. 2013 Jun 21;8(6):e67457. doi: 10.1371/journal.pone.0067457 (PMC3689735; doi:10.1371/journal.pone.0067457)
Supplement: Table S1 — Voxel-based morphometry analyses showing significant correlations between grey matter intensity and recognition of happiness on the Ekman 60 and Ekman Caricatures tasks. Note. All results uncorrected at p<.001; only clusters with at least 100 contiguous voxels are reported. All clusters reported t>3.95. MNI = Montreal Neurological Institute. No clusters greater than 100 voxels were significantly associated with surprise recognition on the Ekman 60 or Ekman Caricatures task. Age included as a nuisance variable for all contrasts. (DOCX) [file pone.0067457.s001.docx]

| **Regions** | | **Hemisphere** | **MNI Coordinates** | | | **Number of Voxels** |
| --- | --- | --- | --- | --- | --- | --- |
|  | |  | **X** | **Y** | **Z** |  |
| **HAPPINESS** | |  |  |  |  |  |
|  | **Ekman 60** |  |  |  |  |  |
| Planum polare, extending into insula | | Left | -44 | -12 | -12 | 247 |
| Precuneus, posterior cingulate | | Right | 2 | -50 | 46 | 162 |
| Superior temporal gyrus, planum polare | | Right | 68 | -22 | 14 | 141 |
|  | **Caricatures** |  |  |  |  |  |
| Orbitofrontal cortex | | Right | 52 | 30 | -14 | 184 |
| Superior parietal lobule | | Right | 14 | -50 | 66 | 121 |
